# Case Study of Traumatised Sub-Postmasters

**DOI:** 10.1192/bjo.2024.661

**Published:** 2024-08-01

**Authors:** Paul Foster

**Affiliations:** Psychiatric & Psychological Consultant Services, London, United Kingdom

## Abstract

**Aims:**

The scandal of sub-postmasters wrongly accused by the post office of offences relating to the faulty IT Horizon system is of ongoing media prominence.

Since May 2021 I have undertaken personal injury medico legal assessments at the request of a solicitor representing those falsely accused and convicted of offences by the post office. Their convictions had been quashed in the court of appeal.

**Methods:**

I interviewed fourteen individuals, age range 35 to 70 years, five women and nine men. Ten had brought claims for Malicious Prosecution. The remaining four sub-postmasters were part of the historical financial shortfalls scheme set up for those who had not been prosecuted but were applying for compensation.

Of the ten convicted sub-postmasters, four spent periods in prison and the remaining six were given suspended or community sentences. Those in prison were often separated from young children by distance or withholding family members.

I assessed to what extent and in what way their mental health and that of their families had been adversely affected as a result of their experiences.

Assessments were undertaken remotely, including speaking with a family member.

ICD 10 diagnostic criteria were used.

**Results:**

All of the cases revealed evidence of psychopathology at the time of the allegations or convictions and continued to varying degrees subsequently.

A diagnosis of PTSD was made in five cases, Adjustment Disorder in 2 cases, Dysthymia in one case and depressive illness in eight cases. In only four cases had the disorders resolved.

Four of the five cases of PTSD had evidence of a past psychiatric history prior to becoming sub-postmasters. Past history included depression, impulsive overdose, eating disorder, problems related to gambling and abuse of alcohol and cocaine.

Both sub-postmasters who had been accused of taking money due to faulty software and those who had been wrongly convicted had high rates of psychopathology.

These findings are consistent with the only other study of the psychological effects on sub-postmasters which found high rates of psychopathology in both accused and convicted individuals.

Existing diagnostic criteria were limited in capturing the suffering of individuals who had endured such complex trauma so a narrative description including the effects on family members was also used.

**Conclusion:**

This study of the mental health of falsely accused sub-postmasters demonstrates a high degree of psychopathology which may require therapeutic intervention.

